# 

**Published:** 1824-03-01

**Authors:** 


### Contents

OF

No. 16, Vol. IV. for MARCH, 1824.

Art. I.

M. Tacheron's Pathological Researches - - - - - - 743

II.

TRANSACTIONS OF THE SURGEON APOTHECARIES.

Art. 1. Mr. Shillito's Case of Spontaneous Rupture of the Uterus, in the Seventh Month of Pregnancy - - 762
2. Mr. C. T. Haden's Cases of Poisoning - - - - 765
3. Mr. Powell's Case of Injury to the Upper Jaw - - 766
4. Mr. Rodd on Belladonna externally - - - - - 766
5. Mr. Hunter's Case of Aortic Aneurism - - - - 767
6. Dr. Kerrison's Communications - - - - - - 767
7. Mr. Evans' Collection of Mal-Practices - - - - 768
8. Mr. Evans' Case of Poisoning, &c. - - - - - - 768
9. Mr. Sandwith on Venous Congestion - - - - - 769
10. Mr. Newnham's Case of Unusual Utero-Gestation 771
11. Dr. Wallis on Colchicum Autumnale - - - - - 771
12. Mr. Haden on Lameness of the Foot - - - - 772
13. Mr. Haden (of Derby) on Club-Feet - - - - 773
14. Memoir on the late Mr. Morel - - - - - - 773

III.

Reports of the Glasgow Asylum for Lunatics - - - - - 775

IV.

MEDICO-CHIRURGICAL TRANSACTIONS, VOL. XII. PART II.

1. Mr. Brayne on Biliary Calculi - - - - - - - - 791
2. Mr. Henry Earle on Local Irritation- - - - - - - 793
———— on Diseases of the Lips - - - - - 794
———— on Affections of the Face - - - - 796
———— on Affections of the Prepuce- - - - 797
———— on Chimney-Sweepers' Cancer - - - 799
3. Mr. Hammond on Destruction of the Fœtal Brain - - - 800
4. Dr. Roots on Bronchocele - - - - - - - - - 801
5. Dr. Kerrison on Dilatation of the Urethra - - - - - 802
6. Sir Astley Cooper on Extraction of Calculi - - - - - 803
7. Dr. Gregory on Small Pox post Vaccinam - - - - - 806

V.

Dr. Marshall Hall on Semeiology - - - - - - - - - - 811

VI.

Mr. Pearson's Life of Mr. Hey - - - - - - - - - - - 827

VII.

PHRENOLOGY.

1. Transactions of the Phrenological Society of Edinburgh
2. Phrenological Journal of Edinburgh - - - - - - - 847
Art. 1. Mr. George Combe's Preliminary Discourse - - - 848
2. Dr. Poole's View of Spurzheim's Lectures - - - 851
3. Mr. Scott on Various Functions and Faculties- - - 854
4. Mr. Coombe on Injuries of the Brain, &c. &c. &c. - 855
5. Answers to Dr. Barclay's Remarks on Phrenology - 868
6. Dr. Patterson on the Phrenology of the Hindoos - - 874
7. The New Phrenological Journal of Edinburgh - - 876

VIII.

Mr. Shaw on Spinal Distortions - - - - - - - - - - 882

IX

Quarterly Periscope,

OR,

*SPIRIT OF THE PUBLIC JOURNALS.*

Remarks on the Increase of Medical Journals - - - - - - 911

I. PATHOLOGY.

1. M. Andral on Diaphragmatic Pleuritis - - - - - - - 912
2. Dr. Gregory's Case of Encysted Tumour - - - - - - 915
3. On the Fever in the Bann and at Ascension - - - - 916
4. M. Magistel on Hydrophobia - - - - - - - - - - 917
5. On Delirium Tremens vel Ebriositatis - - - - - - - 918
6. Scirrhous Cæcum mistaken for Aneurism - - - - - - 919
7. Sudden Death with fallacious Symptoms - - - - - - 920
8. Dr. Davy on Pneumo-Thorax - - - - - - - - - - 922
9. Cases of, and Remarks on Blue Disease - - - - - - 924
10. Dr. Prichard on Paralysis - - - - - - - - - - - 926
11. Dr. Robson on Cholera Epidemica - - - - - - - - 927
12. Mr. Denton on Hæmatemesis - - - - - - - - - - 928
13. Dr. Forbes on Pneumonia - - - - - - - - - - - 928
14. Mr. Gaitskell on Phthisis Pulmonalis - - - - - - - 929
15. On Concussion of the Brain, by Mr. Shipman - - - - 929
16. Case of Internal Hæmorrhage, Mr. Lloyd - - - - - - 930
17. Mr. Pretty's Case of Hydatid simulating Ascites - - 930
18. Expulsion of a Portion of Intestine per Anum - - - - 932
19. Tetanus - - - - - - - - - - - - - - - - - - - 933

20. On the Treatment of Acute Rheumatism - - - - - - 933
21. Mr. Swallow on Carbuncle - - - - - - - - - - - 934
22. Physiological Pathology - - - - - - - - - - - - 934
23. Dr. Carter on Diabetes - - - - - - - - - - - - 935

### II. THERAPEUTICS.

1. Injection of Opium into the Veins - - - - - - - - - 935
2. Dr. Maxwell on Constipation of the Bowels - - - - - 937
3. Mr. Sprague's Pharmaceutical Formulæ - - - - - - 938
4. Corrector of Putrefaction - - - - - - - - - - - 939
5. Opium in Acute Mania - - - - - - - - - - - - 939
6. Prussic Acid in Dyspepsia - - - - - - - - - - - 940
7. Leeches to Internal Surfaces - - - - - - - - - - 940
8. Obstruction of the Bowels, Remedy for - - - - - - 941
9. Mode of Arresting Salivation - - - - - - - - - - 941
10. Purpura Hæmorrhagica - - - - - - - - - - - - 941
11. Bloodletting in Hydrothorax - - - - - - - - - - 942
12. Supposed Hydrophobia - - - - - - - - - - - - 942
13. Strychnine and Brucine - - - - - - - - - - - - 944
14. Degeneration of Muscle - - - - - - - - - - - - 945

### III. SURGERY.

Surgical Operations - - - - - - - - - - - - - - 945
1. Œsophagotomy - - - - - - - - - - - - - - - 945
2. Wry-Neck - - - - - - - - - - - - - - - - - 946
3. Amputation at the Hip Joint - - - - - - - - - - 947
4. Amputation, General Remarks on - - - - - - - - 949
4. (bis) Rupture of the Axillary Artery - - - - - - - 951
5. Extravasation within the Cranium - - - - - - - - 952
6. Fracture of the Cranium (Mr. Baker) - - - - - - - 953
7. Tourniquets in Amputation - - - - - - - - - - - 953
8. Ligature of the Subclavian Artery - - - - - - - - 954
9. M. Richerand on Varicose Veins - - - - - - - - - 954
10. On the Diagnosis of Fractures by the Stethoscope - - 954
11. Remarkable Case of Tracheotomy - - - - - - - - 955
12. Case of Spontaneous Cure of Femoral Aneurism - - - 955
13. On Elongation of the Uvula - - - - - - - - - - 956
14. Acupuncture (Mr. Churchill) - - - - - - - - - - 956
15. Mr. Liston's Case of remarkable Tumour removed - 957
16. Dr. Tweedale and Mr. Finch on Acupuncture - - - 958
17. Operation for Subclavian Aneurism - - - - - - - 958
18. Suppression of Urine - - - - - - - - - - - - - 959
19. Retention of Urine - - - - - - - - - - - - - - 959
20. Taliacotian Operation - - - - - - - - - - - - - 961

### IV. MIDWIFERY.

1. Dr. Douglas on Extraction of the Placenta - - - - - 961
2. Obstetric delinquency - - - - - - - - - - - - - 961
3. Dr. Cumin on Vesico-Vaginal Fistula - - - - - - - 962

4. Dr. H. Davies on Puerperal Convulsions - - - - - - 963

5. Cæsarean Operation - - - - - - - - - - - - - - 964

6. Division of the Symphysis Pubis - - - - - - - - - 965

V. PHYSIOLOGY.

1. Physiology of the Urinary Secretion - - - - - - - - 965
2. The Circulation of the Blood - - - - - - - - - - - 966
3. Physiology of the Nervous System - - - - - - - - - 966

VI. LEGAL MEDICINE.

1. Dr. Castaing's Case considered - - - - - - - - 968
2. Forensic Phrenology, curious Case of - - - - - - - 969
3. Homicide, curious Trial for - - - - - - - - - - - 969
4. Peritonitis resembling the Effects of Poison - - - - 970
5. Poisoning by Nux Vomica - - - - - - - - - - - 972
6. Infanticide, Trial for - - - - - - - - - - - - - - 972

X.

The Lancet; or the Property and Privileges of Lecturers, Medical Societies, and Hospitals considered - - - - - - 973

XI.

EXTRA-LIMITES.

1. Dr. Musgrave on the Question of Contagion in Yellow Fever, in Answer to Sir Gilbert Blane - - - - - - 979
2. Dr. William Burnett on Mercurial Vapour - - - - - 1010

XII.

Bibliographical Record - - - - - - - - - - - - - - 1015

XIII.

Miscellaneous Intelligence, &c. &c. - - - - - - - - - 1019
List of Additional Subscribers - - - - - - - - - - - 1022

